# Predicting peptides binding to MHC class II molecules using multi-objective evolutionary algorithms

**DOI:** 10.1186/1471-2105-8-459

**Published:** 2007-11-22

**Authors:** Menaka Rajapakse, Bertil Schmidt, Lin Feng, Vladimir Brusic

**Affiliations:** 1 Institute for Infocomm Research, 21 Heng Mui Keng Terrace, 119613 Singapore; 2 NICTA VRL, University of Melbourne, Parkville, 3010 Australia; 3 School of Computer Engineering, Nanyang Technological University, Block N4, Nanyang Avenue, 639798 Singapore; 4 Cancer Vaccine Center, Dana-Farber Cancer Institute, Harvard Medical School, Boston, MA 02115 USA

## Abstract

**Background:**

Peptides binding to Major Histocompatibility Complex (MHC) class II molecules are crucial for initiation and regulation of immune responses. Predicting peptides that bind to a specific MHC molecule plays an important role in determining potential candidates for vaccines. The binding groove in class II MHC is open at both ends, allowing peptides longer than 9-mer to bind. Finding the consensus motif facilitating the binding of peptides to a MHC class II molecule is difficult because of different lengths of binding peptides and varying location of 9-mer binding core. The level of difficulty increases when the molecule is promiscuous and binds to a large number of low affinity peptides.

In this paper, we propose two approaches using multi-objective evolutionary algorithms (MOEA) for predicting peptides binding to MHC class II molecules. One uses the information from both binders and non-binders for self-discovery of motifs. The other, in addition, uses information from experimentally determined motifs for guided-discovery of motifs.

**Results:**

The proposed methods are intended for finding peptides binding to MHC class II I-A^g7 ^molecule – a promiscuous binder to a large number of low affinity peptides. Cross-validation results across experiments on two motifs derived for I-A^g7 ^datasets demonstrate better generalization abilities and accuracies of the present method over earlier approaches. Further, the proposed method was validated and compared on two publicly available benchmark datasets: (1) an ensemble of qualitative HLA-DRB1*0401 peptide data obtained from five different sources, and (2) quantitative peptide data obtained for sixteen different alleles comprising of three mouse alleles and thirteen HLA alleles. The proposed method outperformed earlier methods on most datasets, indicating that it is well suited for finding peptides binding to MHC class II molecules.

**Conclusion:**

We present two MOEA-based algorithms for finding motifs, one for self-discovery and the other for guided-discovery by experimentally determined motifs, and thereby predicting binding peptides to I-A^g7 ^molecule. Our experiments show that the proposed MOEA-based algorithms are better than earlier methods in predicting binding sites not only on I-A^g7 ^but also on most alleles of class II MHC benchmark datasets. This shows that our methods could be applicable to find binding motifs in a wide range of alleles.

## Background

Major histocompatibility complex (MHC) molecules play a key role in initiating immune responses. They bind to and expose an antigen (or short peptides) to T cell receptors (TCR) triggering an immune response against the infected cell or foreign agent. MHC molecules make multiple contacts with the side-chains of binding peptides, which define the binding motif and determine the specificity of binding [1]. Prediction of peptides binding to a MHC class II molecule is difficult due to different types of side chains and because the length of the binding peptides is longer than 9aa (approximately 11 to 22aa) [1,2]. It has been previously observed that a core of 9aa is sufficient for binding peptides to a MHC class II molecules [3], however, the exact location of the binding core (or motif) within the peptide is usually unknown and vary.

A binding motif is usually represented either by a consensus sequence or as a weight matrix [4]. The presence or composition of a motif can be experimentally determined from a large pool of putative binding peptides [3,5]. However, such wet-lab experiments are costly, time consuming, and cumbersome. Amino acids at specific sites of a motif, contributing significantly to the binding are referred to as *primary anchor residues *and the corresponding sites as *anchor positions*. By using such position-specific information, earlier studies have found weight matrix models elaborating the nature and strength of binding motifs [6,7]. These models offer binding strengths of every residue at specific sites in the form of a position specific scoring matrix (PSSM).[7]

In general, MHC class-II prediction methods are categorized into two main classes [8]: (1) quantitative prediction methods that predict inhibitory concentration (IC_50_) values and (2) qualitative prediction methods that determine the binding status (binder or non-binder) based on the predictive score. Recent quantitative prediction approaches include SVRMHC [8], PLS-ISC [9], ARB [10], and SMM-align [11]. The ARB approach uses full length of the peptide whereas both SVRMHC and PLS-ISC approaches use a preprocessing step involving alignment of sequences, based on anchor position-specific residues. The underlying assumption of SMM-align is that amino acids occupying the 9-mer binding core motif are sufficient to determine the affinity of peptide-MHC binding. However, in some cases, the predictive performance could be improved by incorporating terminal residues known as peptide flanking residues (PFR) [11].

Qualitative prediction approaches use classifiers such as artificial neural networks [12-16], hidden Markov models [4,17], support vector machines [18-21], and their hybrids [22], or profile analysis such as those using iterative learning [23-26], stochastic approaches (MEME) [27,28], Gibbs motif sampler [29-32], profile motifs (RANKPEP) [33,34], DNA microarrays and virtual matrices (TEPITOPE) [35], and evolutionary algorithms (EA) [36]. However, given a set of sequences of differing lengths with known binding affinities, the location of the binding core within each sequence must be first identified before classification of sequences. Classical multiple sequence alignment techniques often fail to detect binding cores in MHC class II binding peptides because of weak instances of binding motifs.

All methods predicting peptides binding to MHC molecules have their pros and cons; most show good performance only for datasets upon which they were developed. Therefore, there is a need for new algorithms that perform well on previously unseen data. We propose to use MOEA to align a set of experimentally determined binding peptides at their binding cores and subsequently derive the consensus motif. The methods are especially useful when molecules are promiscuous and bind to a large number of low affinity peptides. The preliminary results of our work have been presented in [37].

I-A^g7 ^is the MHC class II molecule of the NOD mouse, critical for the development of insulin-dependent diabetes mellitus (IDDM) and other autoimmune disorders [38-43]. Knowledge of peptides binding to I-A^g7 ^is important in understanding the molecular basis of development of IDDM in NOD mice. Experiments have demonstrated that I-A^g7 ^binding peptides are 9–30aa long [44]. Finding motifs in peptide binding to I-A^g7 ^is a non-trivial problem [45,46]. Despite numerous attempts, no consensus has been reached on the rules of peptide binding to I-A^g7 ^molecule [38-48]. However, computational analyses on multiple datasets indicate that experimental motifs satisfy only a subset of rules describing the optimal motif.

To demonstrate the utility in predicting peptides binding to other MHC molecules, our method is tested on two benchmark datasets comprising of peptides of number of different HLA (human MHC) and mouse alleles. The first dataset, referred to as BM-Set1 here onwards, consists of different combinations of peptides of HLA-DRB1*0401 allele, and the second dataset, BM-Set2, consists of datasets from thirteen different HLA alleles and three mouse alleles.

### Multi-Objective Evolutionary Algorithms (MOEA)

Evolutionary algorithms (EA) are based on the principles of biological evolution and have often been successful in solving complex search and optimization problems. Majority of bioinformatics applications of EA have been in the discovery of motifs such as transcription factor binding sites [49-53]. Yet, only a few researchers have used EA for the prediction of peptides binding to protein sequences [36].

An EA consists of (1) representing input variables as individuals or chromosomes (binary or real valued) in a population, (2) formulating the fitness (objective function) to evaluate individuals, (3) generating a new population by genetic operations (such as reproduction, crossover, and mutation) on the current population, and (4) determining if the population has reached the optimal fitness. The algorithm begins with an initial population and evolves over time. At a particular instance of evolution, every individual is evaluated by its fitness. New populations (offspring) are produced from highly fit individuals (parents) selected, which undergo genetic operations. Each offspring is paired and compared to its parents. Highly fit individuals are retained in the population while less fit individuals are discarded. Search mechanisms such as elitism, constraint-handling, and multi-objective optimization are available for finding a better spread of solutions, depending on the needs of the optimization problem [54-57].

Multi-objective evolutionary algorithms (MOEA) are used to solve problems which require simultaneous optimization of a number of competing objective functions [58-61]. MOEA maintains a set of solutions ranked by their dominance at a given instant of the evolution. A solution is said to dominate another if it is better or equal with respect to all objectives and strictly better in at least one objective [58]. Often, there are more than one non-dominated solutions, representing the best ones, collectively known as the *Pareto *front. MOEA algorithms result in a *Pareto optimal set *of solutions.

Non-dominated Sorting Genetic Algorithm II (NSGA-II) was recently introduced to incorporate several new genetic mechanisms for better convergence, such as non-dominated sorting, elitism, diversity preservation, and constraint handling [58]. In NSGA-II, a population is subjected to several rounds of non-dominated sorting. That is, all the non-dominated individuals are identified and assigned the same fitness value until a new set of non-dominated solutions is found. The solutions found in subsequent rounds are assigned fitness values lower than those in the previous rounds. This process continues until the whole population is partitioned into non-dominated fronts with diverse fitness values. The elitism prevents the loss of fit individuals encountered in earlier generations by allowing earlier solutions to survive in the subsequent generations. The diversity of Pareto-optimal solutions is maintained by imposing a measure referred to as *crowding distance*. A solution that satisfies the constraints defined by the objective functions is called a *feasible solution*.

### Peptide Binding to MHC Class II I-A^g7^

In this paper, we attempt to find an optimal motif describing peptide binding to MHC class II molecules, using experimentally determined binding data. There are several factors that impede the derivation of such a consensus motif. The first is the strong resemblance among the peptides isolated in a single experiment and the second is the diversity among different datasets. A motif derived from a dataset lacking diversity indicates a bias towards the dataset used in deriving the motif. Such motifs are difficult to generalize on other experimental or previously unseen datasets. The MOEA based motif detection algorithm is designed to find a consensus motif on I-A^g7 ^datasets, which alleviates the influences arising from biased datasets and thereby predicts binding peptides more accurately in new datasets.

## Results

### Predicting Peptides Binding to MHC Class II

We use our approach to find a consensus motif on seven experimental datasets of peptides binding to I-A^g7 ^molecules, obtained from literature [40-43,62-64]. The motif is validated using an independent testing set generated from the Stratmann dataset [46]. The overall quality of prediction was measured using area under curve (AUC) of the receiver operating characteristics (ROC) curve [65-67]. AUC values of all feasible solutions in the final population of EA were evaluated and the solution with the highest AUC was chosen as the consensus motif (see Additional file 1).

Table 1 shows the information of the datasets extracted from literature, which were used in the training. A blank '-'indicates the unavailability of a particular information. As an example, the details of the experimental motif of Reizis *et al *are given in Table 2. Table 3 shows the performance when an experimental motif is used to predict peptide binders in other datasets. As seen, a motif of a particular experiment does not characterize peptide binding of I-A^g7 ^molecules in other datasets. Table 4 shows the cross-validation performance of two motifs (by self-discovery and guided-discovery) derived using MOEA; in a particular cross-validation run, one experimental dataset was excluded and the motif was derived using the information of the remaining datasets. The motif was tested for predicting binders and non-binders of the left-out dataset. The self-discovery approach uses only the binding information whereas the guided-discovery uses both binding information as well as information associated with experimental motifs. As seen in Table 4, by achieving AUC values greater than 0.7 for all cross-validation runs, MOEA derived motifs demonstrate better generalization capabilities compared to experimentally determined motifs. The binding motifs derived from self-discovery and guided-discovery are illustrated as sequence logo plots [68] in the Additional file 2.

**Table 1:** I-A^g7 ^datasets and experimental motifs

Dataset	Experimental Motif	Non-binders	Binders	Reference
Reizis	*m*(Reizis)	21	33	[40]
Harrison	*m*(Harrison)	19	157	[41]
Gregori	*m*(Gregori)	31	109	[43]
Latek	*m*(Latek)	8	37	[42]
-	*m*(Rammensee)	-	-	[44]
-	*m*(Reich)	-	-	[38]
-	*m*(Amor)	-	-	[39]
Corper	-	35	13	[62]
MHCPEP	-	-	176	[63]
Yu	-	16	10	[64]
Brusic	-	37	-	[unpublished]

Information on I-A^g7 ^related peptide binding datasets and motifs. Unavailable information is indicated by "-".

**Table 2:** Representation of an experimentally derived I-A^g7 ^motif

Position	Well-Tolerated	Weakly-Tolerated	Non-Tolerated
P1	VEQMHLPD	-	R
P2	-	-	-
P3	-	-	-
**P4**	**ILPV**	**HY**	**QEK**
P5	-	-	-
**P6**	**ATSNV**	**-**	**LYQK**
P7	QVYLHINRF	-	-
P8	-	-	-
**P9**	**ED**	**SM**	**LYTQK**

The description of experimentally determined I-A^g7 ^9-mer peptide binding motif by Reizis: each position accommodates a well-tolerated, weakly-tolerated, or non-tolerated amino acid. The positions P4, P6 and P9 are the primary anchor positions where binding is highly likely to occur.

**Table 3:** Validation of I-A^g7 ^experimental motifs

Experimental Motif	AUC value						
	
	Datasets						
	
	Reizis	Harrison	Gregori	Latek	Corper	MHCPEP	Yu
*m*(Reizis)	0.95	0.68	0.74	0.95	0.50	0.59	0.48
*m*(Harrison)	0.75	0.88	0.69	0.64	0.53	0.72	0.33
*m*(Gregori)	0.64	0.68	0.71	0.73	0.40	0.64	0.61
*m*(Latek)	0.66	0.72	0.80	0.95	0.64	0.52	0.75
*m*(Rammensee	0.49	0.64	0.76	0.82	0.60	0.48	0.43
*m*(Reich)	0.55	0.64	0.69	0.58	0.56	0.47	0.50
*m*(Amor)	0.69	0.54	0.66	0.70	0.56	0.66	0.40

Performance measured by AUC of experimentally determined I-A^g7 ^motifs on their own datasets and other experimental datasets.

**Table 4:** Performance of I-A^g7 ^MOEA derived motifs

	AUC value						
	
MOEA-derived Motifs	Datasets						
	
	Reizis	Harrison	Gregori	Latek	Corper	MHCPEP	Yu
self-discovery	0.75	0.75	0.77	0.93	0.70	0.75	0.75
guided-discovery	0.77	0.74	0.81	0.83	0.72	0.77	0.71

Seven-fold cross-validation accuracies of MOEA derived motifs on training dataset.

**Figure 1:**
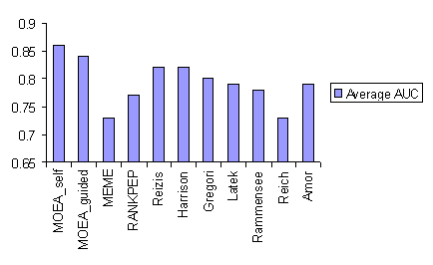
**Comparison of Performances**. Comparison of performance of MOEA based algorithms – self-discovery and guided-discovery – against MEME, RANKPEP, and experimental motifs on the balanced I-A^g7 ^test datasets (the performance was averaged over 25 test datasets)

To compare the performance of our method with earlier methods, a training dataset was created by combining all the experimental datasets given in Table 1. Motifs derived on the training dataset were tested on an independent test dataset – a balanced set generated from Stratmann dataset. The Stratmann dataset was balanced by adding randomly generated non-binders. Twenty five such balanced test datasets were assembled by generating random samples starting from different seeds and adding them to the Stratmann dataset. The results reported are based on the average AUC values over all balanced test sets. Figure 1 shows comparison of performances of motifs derived by MOEA and by earlier motif prediction approaches such as MEME and RANKPEP. An increase of 4–10% in predictive performance is observed with MOEA over the other approaches.

Comparison of performances of MOEA derived motifs for BM-Set1 (see Table 5) with enhanced Gibbs sampler [32], TEPITOPE [35], SVRMHC [8] and ARB [10], is given in Table 6. As seen, MOEA shows comparable or superior performance with Gibbs sampler on all datasets except for the Southwood dataset. Out of the ten non-redundant (NR) datasets, the MOEA outperformed Gibbs sampler, TEPITOPE, SVRMHC and ARB by seven, nine, eight and ten datasets, respectively.

The performance of MOEA on BM-Set2 (see Table 7) was compared with Gibbs sampler [32], TEPITOPE [35], SVRMHC [8], ARB [10] and NetMHCII [11]. Each allele dataset was subjected to five-fold cross-validation and the results are given in Table 8. The present method shows comparable or superior performance on majority of allele datasets compared to Gibbs sampler, SVRMHC, TEPITOPE, and NetMHCII. A fair comparison of ARB method cannot be drawn because the method has been trained on quantitative data obtained from IEDB [10].

**Table 5:** Description of peptides in BM-Set1

BM-Set1	Original		NR	
DRB1*0401	Binders	Non-binders	Binders	Non-binders

Set1	694	323	248	283
Set2	381	292	161	255
Set3a	373	217	151	204
Set3b	279	216	128	197
Set4a	323	323	120	283
Set4b	292	292	120	255
Set5a	70	47	65	45
Set5b	48	37	47	37
Southwood	16	6	15	6
Geluk	22	83	19	80

The number of binders and non-binders in the original and non-redundant (NR) datasets in BM-Set1.

**Table 6:** Comparison of performance on BM-Set1

Dataset		AUC				
		
		†SVRMHC	Gibbs	ARB	TEPITOPE	MOEA
Original	set1	0.711	0.799	0.666	0.760	0.760
	set2	0.652	0.766	0.653	0.736	0.765
	set3a	0.626	0.740	0.652	0.730	0.733
	set3b	0.618	0.751	0.666	0.750	0.752
	set4a	0.706	0.788	0.668	0.748	0.748
	set4b	0.664	0.770	0.661	0.748	0.770
	set5a	0.553	0.604	0.539	0.653	0.777
	set5b	0.606	0.621	0.579	0.679	0.748
	Southwood	0.912	0.862	0.514	0.490	0.784
	Geluk	0.697	0.723	0.682	0.710	0.786
NR	set1	0.619	0.673	0.572	0.594	0.587
	set2	0.581	0.665	0.640	0.653	0.685
	set3a	0.578	0.598	0.600	0.598	0.660
	set3b	0.577	0.692	0.669	0.699	0.713
	set4a	0.597	0.671	0.575	0.573	0.599
	set4b	0.577	0.669	0.651	0.655	0.690
	set5a	0.544	0.601	0.536	0.646	0.790
	set5b	0.593	0.610	0.572	0.671	0.743
	Southwood	0.917	0.850	0.671	0.505	0.770
	Geluk	0.655	0.697	0.510	0.670	0.768

Comparison of AUC values of the BM-Set1 (DRB1*0401). †These values are based on smaller dataset sizes as SVRMHC didn't predict values for some of the peptides. The values from the Gibbs sampler were estimated from the matrix provided by the authors in [32].

**Table 7:** Description of peptides in BM-Set2

Type	Allele	Binders	Non-binders
Mouse	I-Ab	43	33
	I-Ad	56	286
	I-As	35	91
HLA	DRB1-0101	920	283
	DRB1-0301	65	409
	DRB1-0401	209	248
	DRB1-0404	74	94
	DRB1-0405	88	83
	DRB1-0701	125	185
	DRB1-0802	58	116
	DRB1-0901	47	70
	DRB1-1101	95	264
	DRB1-1302	101	78
	DRB1-1501	188	177
	DRB4-0101	74	107
	DRB5-0101	112	231

The number of binders and non-binders in each of the dataset in BM-Set2. The datasets in BM-Set2 were obtained from [77]. The DRB3-0101 allele dataset was excluded from the performance comparison due to significant imbalance in the dataset (3 binders and 99 non-binders).

**Table 8:** Comparison of Performance on BM-Set2

Type	Allele	AUC					
		
		SVRMHC	Gibbs	ARB	TEPITOPE	NetMHCII	MOEA
Mouse	I-A^b^	-	-	0.662	-	0.908	0.919
	I-A^d^	-	-	0.819	-	0.818	0.855
	I-A^s^	-	-	-	**-**	0.898	0.889
HLA	DRB1-0101	0.623	0.676	0.666	0.647	0.716	0.651
	DRB1-0301	-	0.722	0.799	0.734	0.765	0.778
	DRB1-0401	0.739	0.759	0.737	0.754	0.758	0.725
	DRB1-0404	-	0.743	0.788	0.829	0.785	0.786
	DRB1-0405	0.701	0.724	0.724	0.790	0.735	0.756
	DRB1-0701	-	0.695	0.749	0.768	0.787	0.735
	DRB1-0802	-	0.721	0.803	0.769	0.756	0.773
	DRB1-0901	-	0.734	0.711	-	0.775	0.712
	DRB1-1101	-	0.715	0.727	0.710	0.734	0.759
	DRB1-1302	-	0.716	0.917	0.720	0.818	0.820
	DRB1-1501	0.730	0.672	0.792	0.726	0.736	0.743
	DRB4-0101	-	0.742	0.800	-	0.736	0.759
	DRB5-0101	0.649	0.618	0.677	0.653	0.664	0.660

Comparison of AUC values from five-fold cross-validation of allele datasets given in BM-Set2. "-" indicates that the allele is unavailable for testing with the respective prediction method.

## Discussion

We proposed two approaches using MOEA for deriving motifs (1) when the information of only the binders and non-binders are known (i.e., self-discovery) and (2) when, in addition, the information of experimentally (wet-lab) determined motifs are available (i.e., guided-discovery).

Since I-A^g7 ^molecule is known to bind to a large number of peptides of low affinity and appears to be a promiscuous binder, the prediction of peptides binding to I-A^g7 ^molecule has been nontrivial. This has lead to the definition of a number of suboptimal consensus motifs specific to the datasets. MOEA derived motifs had superior generalization capabilities to those derived with MEME and RANKPEP techniques as well as to the experimentally determined motifs on other datasets. The performances evaluated on two benchmark datasets indicate that the present MOEA based algorithm is applicable in deriving motifs on other class II MHC alleles as well.

The likelihood of finding an optimal motif by MOEA is higher than by a local or greedy search because of the stochastic nature of EA. The proposed approach learns from the characteristics of both binders and non-binders in the training set whereas other methods use information only from binders to determine motifs [27,32]. Moreover, ranges of the parameters involved in MOEA are known, so the parameters of the fitness functions are quickly estimated in a few cross-validation runs. Furthermore, unlike the earlier methods, the present method does not rely on any prior information such as anchor positions to obtain an alignment, prior distributions, etc., [8,9]. Given sufficient data samples representing both binders and non-binders, the method could be applicable to find motifs in other types of molecules. A future direction of this research would be to integrate additional information such as peptide length [69] and PFR [70] as such information has been shown to have the potential to enhance motif detection [11,69]. This would lead to further improvement of the performance of the present algorithm.

Even though EAs are generally known to be computationally intensive, training for derivation of scoring matrices can be performed off-line and the prediction engines can be provided through web services. As seen in Tables 6 and 8, a single method does not always perform well on all types of allele datasets. Nevertheless, the present method showed higher accuracy in detecting motifs on majority of MHC alleles in the benchmark datasets. Therefore, we believe that MOEA-based methods could provide a general framework for efficiently determining motifs in a wide range of MHC molecules.

In immunology, accuracy and speed in predicting binding peptides is of paramount importance. Computationally predicted binders do subsequently need to be validated with wet-lab experiments. By using computational predictions as an initial step, high cost involved in initial screening and time-consuming clinical testing can be significantly reduced. Towards this end, the proposed MOEA methods present a promising way to predict peptides that bind to MHC class II alleles including promiscuous and low affinity peptide binders.

## Conclusion

We present two MOEA-based algorithms for finding motifs, one for self-discovery and the other for guided-discovery by experimentally determined motifs, and thereby predicting binding peptides to I-A^g7 ^molecule. Our experiments show that the proposed MOEA-based algorithms are better than earlier methods in predicting binding sites not only on I-A^g7 ^but also on most alleles of class II MHC benchmark datasets. This demonstrates the applicability of our methods to find binding motifs in a wide range of MHC alleles.

## Methods

### Datasets

Several I-A^g7 ^datasets were extracted from literature [40-43,62-64] and from Brusic, V.(unpublished data). The numbers of binders and non-binders in each dataset are given in Table 1. The datasets consist of short peptides ranging from 9–30aa in length. Their binding affinities had been experimentally determined by independent studies and classified as binders or non-binders based on IC_50 _values according to the following scheme [41]: good binder (IC_50 _= 100 nM); weak binder (IC_50 _= 2000 nM); non-binder (IC_50 _= 50000 nM). The datasets in [40-43,62-64] were combined into a single training dataset and curated by removing duplicates and redundancy as follows: if a binder is a subsequence of another binder sequence, the longer binder sequence is discarded; if a non-binder is a subsequence of another non-binder, the shorter subsequence is discarded. Let the curated whole dataset be referred to as *training *dataset here onwards and it be denoted by *D *= {(*x*_*i*_, *v*_*i*_): *i *= 1, 2,.... *N*} where *N *is the number of total peptide sequences and *x*_*i *_is the *i*-th peptide sequence with the label *v*_*i *_ε {b, nb} indicating whether the sequence *x*_*i *_is a binder (b) or a non-binder (nb). The number of peptides in the training set *N *= 438 in which the number of binders *N*_b _= 304 and the number of non-binders *N*_nb _= 134.

The set of experimentally validated I-A^g7^motifs [38-44] derived largely from uncorrelated datasets [40-43] was extracted and is illustrated in Table 1 with the distribution of binders and non-binders in each dataset. Table 2 illustrates an experimentally validated motif of I-A^g7 ^reported by Reizis *et al *[40]. Experimental motifs are described by the anchor positions and binding affinities of amino acids of the motif. The residues which contribute significantly to the peptide binding are called primary anchor residues and positions they reside are called anchor positions. An amino acid occupying a specific position within a motif is characterized as well tolerated, weakly tolerated, or non-tolerated based on its involvement in the binding process.

An independent dataset was generated from binders of Stratmann dataset [46], consisting of a diverse set of I-A^g7 ^binding peptides with their binding affinities, to find the test accuracies in predicting binders and non-binders. The Stratmann dataset was balanced with randomly generated 9-mer non-binders so that for testing dataset, *N*_b _= *N*_nb _= 112.

### Binding Score Matrix

A *k*-mer motif of amino acids is characterized by a PSSM *Q *= {*q*_*ia*_}_*k *× 20 _where *q*_*ia *_denotes the binding strength of the site *i *when it is occupied by amino acid *a*. The binding score of a putative motif is computed by adding the binding scores assigned to each amino acid at the respective positions. The binding score indicates the likelihood of the motif binding to the molecule. The binding score *s*_*i *_of sequence *x*_*i *_= (*x*_*i*,1_, *x*_*i*,2_,...*x*_*i*, *n*_) of length *n *is determined by the maximum value of binding scores computed for all *k*-mer subsequences in *x*_*i*_:

si=max⁡j{sij:j=1,2,⋯n−k+1}
 MathType@MTEF@5@5@+=feaafiart1ev1aaatCvAUfKttLearuWrP9MDH5MBPbIqV92AaeXatLxBI9gBaebbnrfifHhDYfgasaacPC6xNi=xI8qiVKYPFjYdHaVhbbf9v8qqaqFr0xc9vqFj0dXdbba91qpepeI8k8fiI+fsY=rqGqVepae9pg0db9vqaiVgFr0xfr=xfr=xc9adbaqaaeGacaGaaiaabeqaaeqabiWaaaGcbaGaem4Cam3aaSbaaSqaaiabdMgaPbqabaGccqGH9aqpdaWfqaqaaiGbc2gaTjabcggaHjabcIha4bWcbaGaemOAaOgabeaakiabcUha7jabdohaZnaaBaaaleaacqWGPbqAcqWGQbGAaeqaaOGaeiOoaOJaemOAaOMaeyypa0JaeGymaeJaeiilaWIaeGOmaiJaeiilaWIaeS47IWKaemOBa4MaeyOeI0Iaem4AaSMaey4kaSIaeGymaeJaeiyFa0haaa@4BBC@

where *s*_*ij *_denotes the binding score of the subsequence beginning at location *j *of the sequence *i*, which is given by

sij=∑l=1,2,⋯kq(j+l),xi(j+l)
 MathType@MTEF@5@5@+=feaafiart1ev1aaatCvAUfKttLearuWrP9MDH5MBPbIqV92AaeXatLxBI9gBaebbnrfifHhDYfgasaacPC6xNi=xI8qiVKYPFjYdHaVhbbf9v8qqaqFr0xc9vqFj0dXdbba91qpepeI8k8fiI+fsY=rqGqVepae9pg0db9vqaiVgFr0xfr=xfr=xc9adbaqaaeGacaGaaiaabeqaaeqabiWaaaGcbaGaem4Cam3aaSbaaSqaaiabdMgaPjabdQgaQbqabaGccqGH9aqpdaaeqbqaaiabdghaXnaaBaaaleaacqGGOaakcqWGQbGAcqGHRaWkcqWGSbaBcqGGPaqkaeqaaOGaeiilaWIaemiEaG3aaSbaaSqaaiabdMgaPjabcIcaOiabdQgaQjabgUcaRiabdYgaSjabcMcaPaqabaaabaGaemiBaWMaeyypa0JaeGymaeJaeiilaWIaeGOmaiJaeiilaWIaeS47IWKaem4AaSgabeqdcqGHris5aaaa@4D17@

and assuming that only one motif instance exists in every sequence, the location j* of the motif is given by

j∗=arg⁡max⁡j{sij:j=1,2,⋯n−k+1}
 MathType@MTEF@5@5@+=feaafiart1ev1aaatCvAUfKttLearuWrP9MDH5MBPbIqV92AaeXatLxBI9gBaebbnrfifHhDYfgasaacPC6xNi=xI8qiVKYPFjYdHaVhbbf9v8qqaqFr0xc9vqFj0dXdbba91qpepeI8k8fiI+fsY=rqGqVepae9pg0db9vqaiVgFr0xfr=xfr=xc9adbaqaaeGacaGaaiaabeqaaeqabiWaaaGcbaGaemOAaO2aaWbaaSqabeaacqGHxiIkaaGccqGH9aqpcyGGHbqycqGGYbGCcqGGNbWzdaWfqaqaaiGbc2gaTjabcggaHjabcIha4bWcbaGaemOAaOgabeaakiabcUha7jabdohaZnaaBaaaleaacqWGPbqAcqWGQbGAaeqaaOGaeiOoaOJaemOAaOMaeyypa0JaeGymaeJaeiilaWIaeGOmaiJaeiilaWIaeS47IWKaemOBa4MaeyOeI0Iaem4AaSMaey4kaSIaeGymaeJaeiyFa0haaa@4F4D@

That is, the most likely motif instance of sequence *x*_*i*_, say *m*_*i*_, is given by the sequence *m*_*i *_= (*x*_ij*_·*x*_ij* + 1_,... *x*_ij* + *k*-1_).

### Self-discovery of Motif

We derive a consensus motif from the training dataset which consists of peptides from several experiments and of varying lengths. The positions of binding cores within the peptides are unknown. The elements of the PSSM are represented as 20*k*-tuples (*q*_*ia*_, : *i *= 1,... *k*; *a ε *Ω) where Ω represents the amino acid alphabet. Each element in the *k*-tuple is converted to a real number representation using a binary word of size *θ *so that *q*_*ia *_∈ [0, 2^*θ*^-1]. The *k*-mer motif is therefore represented by an individual of 20*kθ *long string in the EA. Let the population at *t*-th iteration of the evolution is denoted by *q*(*t*) = {*q*_1_(*t*), *q*_2_(*t*),..... *q*_*M *_(*t*)} where *q*_*j*_(*t*) represents an individual in a population of size *M*.

The fitness function is designed to arrive at an optimal consensus of the motif, by using the training dataset. A solution is evaluated based on its ability to maximize the accuracies in identifying true binders (TP) and true non-binders (TN) as well as to widen the gap between the total score for binders and non-binders. This is achieved by two fitness functions: *f*_1 _to minimize the sum of false positives (FP) and false negatives (FN), and *f*_2 _to minimize the ratio between the average cumulative scores of non-binders and binders:

*f*_1 _= FN + *κ*_1 _FP

f2=NbNnb∑i=1Ns(mi)δ(vi=nb)∑i=1Ns(mi)δ(vi=b)
 MathType@MTEF@5@5@+=feaafiart1ev1aaatCvAUfKttLearuWrP9MDH5MBPbIqV92AaeXatLxBI9gBaebbnrfifHhDYfgasaacPC6xNi=xI8qiVKYPFjYdHaVhbbf9v8qqaqFr0xc9vqFj0dXdbba91qpepeI8k8fiI+fsY=rqGqVepae9pg0db9vqaiVgFr0xfr=xfr=xc9adbaqaaeGacaGaaiaabeqaaeqabiWaaaGcbaGaemOzay2aaSbaaSqaaiabikdaYaqabaGccqGH9aqpjuaGdaWcaaqaaiabd6eaonaaBaaabaGaeeOyaigabeaaaeaacqWGobGtdaWgaaqaaiabb6gaUjabbkgaIbqabaaaamaalaaabaWaaabCaeaacqWGZbWCcqGGOaakcqWGTbqBdaWgaaqaaiabdMgaPbqabaGaeiykaKccciGae8hTdqMaeiikaGIaemODay3aaSbaaeaacqWGPbqAaeqaaiabg2da9iabb6gaUjabbkgaIjabcMcaPaqaaiabdMgaPjabg2da9iabigdaXaqaaiabd6eaobGaeyyeIuoaaeaadaaeWbqaaiabdohaZjabcIcaOiabd2gaTnaaBaaabaGaemyAaKgabeaacqGGPaqkcqWF0oazcqGGOaakcqWG2bGDdaWgaaqaaiabdMgaPbqabaGaeyypa0JaeeOyaiMaeiykaKcabaGaemyAaKMaeyypa0JaeGymaedabaGaemOta4eacqGHris5aaaaaaa@62AE@

Eqs. (4) and (5) are minimized and subjected to following two constraints:

FPNnb≤1α1
 MathType@MTEF@5@5@+=feaafiart1ev1aaatCvAUfKttLearuWrP9MDH5MBPbIqV92AaeXatLxBI9gBaebbnrfifHhDYfgasaacPC6xNi=xI8qiVKYPFjYdHaVhbbf9v8qqaqFr0xc9vqFj0dXdbba91qpepeI8k8fiI+fsY=rqGqVepae9pg0db9vqaiVgFr0xfr=xfr=xc9adbaqaaeGacaGaaiaabeqaaeqabiWaaaGcbaqcfa4aaSaaaeaacqqGgbGrcqqGqbauaeaacqWGobGtdaWgaaqaaiabb6gaUjabbkgaIbqabaaaaOGaeyizImAcfa4aaSaaaeaacqaIXaqmaeaaiiGacqWFXoqydaWgaaqaaiabigdaXaqabaaaaaaa@38F1@

FNNb≤1α2
 MathType@MTEF@5@5@+=feaafiart1ev1aaatCvAUfKttLearuWrP9MDH5MBPbIqV92AaeXatLxBI9gBaebbnrfifHhDYfgasaacPC6xNi=xI8qiVKYPFjYdHaVhbbf9v8qqaqFr0xc9vqFj0dXdbba91qpepeI8k8fiI+fsY=rqGqVepae9pg0db9vqaiVgFr0xfr=xfr=xc9adbaqaaeGacaGaaiaabeqaaeqabiWaaaGcbaqcfa4aaSaaaeaacqqGgbGrcqqGobGtaeaacqWGobGtdaWgaaqaaiabbkgaIbqabaaaaOGaeyizImAcfa4aaSaaaeaacqaIXaqmaeaaiiGacqWFXoqydaWgaaqaaiabikdaYaqabaaaaaaa@378C@

where *s*(*m*_*i*_) denotes the score computed for the most likely motif instance *m*_*i *_of sequence *x*_*i *_of the training dataset, and Kronecker *δ *is one when the argument is satisfied and otherwise is zero. *N*_b _and *N*_nb _are the total counts of binders and non-binders in the dataset. The constant *κ*_1 _(>*N*_b_/*N*_nb _for *N*_b _> *N*_nb_, or vice versa) was empirically determined to minimize the number of false positives. The two parameters *α*_1 _(<<*N*_nb_) and *α*_2 _(<<*N*_b_) are set to minimize FP and FN rates, respectively. If none of the individuals satisfies the above constraints, MOEA reports no feasible solution. Given the training set, a few trial runs with different initializations are necessary to determine the best values of *α*_1 _and *α*_2_.

### Scoring of Experimental Motifs

The description of an experimental *k*-mer motif conveys three kinds of information at each site: (1) the amino acid occupied, (2) the tolerance level of the amino acid, and (3) the strength of binding. Let us denote a *k*-mer motif validated in experiment "e" by *m*(e) and the tolerance level of the residue at site *j *by *ρ*_*j *_where *ρ*_*j *_∈ {well, weak, unknown, non – tolerated}. The binding strength of site *j *is expressed by *σ*_*j *_∈ {primary – anchor, secondary – anchor, other}. Then, the binding score for a *k*-mer experimental motif is given by

s(m(e))=∑j=1kρj⋅σj
 MathType@MTEF@5@5@+=feaafiart1ev1aaatCvAUfKttLearuWrP9MDH5MBPbIqV92AaeXatLxBI9gBaebbnrfifHhDYfgasaacPC6xNi=xI8qiVKYPFjYdHaVhbbf9v8qqaqFr0xc9vqFj0dXdbba91qpepeI8k8fiI+fsY=rqGqVepae9pg0db9vqaiVgFr0xfr=xfr=xc9adbaqaaeGacaGaaiaabeqaaeqabiWaaaGcbaGaem4CamNaeiikaGIaemyBa0MaeiikaGIaeeyzauMaeiykaKIaeiykaKIaeyypa0ZaaabCaeaaiiGacqWFbpGCdaWgaaWcbaGaemOAaOgabeaakiabgwSixlab=n8aZnaaBaaaleaacqWGQbGAaeqaaaqaaiabdQgaQjabg2da9iabigdaXaqaaiabdUgaRbqdcqGHris5aaaa@4482@

### Guided-discovery of Motif

In this algorithm, we assume that experimentally determined motifs are available along with the experimental datasets. An MOEA is proposed to determine a motif closer to experimental motifs. An objective function *f*_3 _is proposed to best represent the characteristics of the motif that is close to the knowledge embedded in the experimental motifs:

f3=∑e|Q^−Q(m(e))|
 MathType@MTEF@5@5@+=feaafiart1ev1aaatCvAUfKttLearuWrP9MDH5MBPbIqV92AaeXatLxBI9gBaebbnrfifHhDYfgasaacPC6xNi=xI8qiVKYPFjYdHaVhbbf9v8qqaqFr0xc9vqFj0dXdbba91qpepeI8k8fiI+fsY=rqGqVepae9pg0db9vqaiVgFr0xfr=xfr=xc9adbaqaaeGacaGaaiaabeqaaeqabiWaaaGcbaGaemOzay2aaSbaaSqaaiabiodaZaqabaGccqGH9aqpdaaeqbqaamaaemaabaGafmyuaeLbaKaacqGHsislcqWGrbqucqGGOaakcqWGTbqBcqGGOaakcqqGLbqzcqGGPaqkcqGGPaqkaiaawEa7caGLiWoaaSqaaiabbwgaLbqab0GaeyyeIuoaaaa@3FA7@

where Q^
 MathType@MTEF@5@5@+=feaafiart1ev1aaatCvAUfKttLearuWrP9MDH5MBPbIqV92AaeXatLxBI9gBaebbnrfifHhDYfgasaacPC6xNi=xH8viVGI8Gi=hEeeu0xXdbba9frFj0xb9qqpG0dXdb9aspeI8k8fiI+fsY=rqGqVepae9pg0db9vqaiVgFr0xfr=xfr=xc9adbaqaaeGacaGaaiaabeqaaeqabiWaaaGcbaGafmyuaeLbaKaaaaa@2D0E@ denotes the estimated PSSM of the motif. We use the same objective function in Eq. (4) to accurately predict binders of the training dataset. The MOEA minimizes the objective functions given in Eqs. (4) and (9), subjected to the two constraints given in Eqs. (6) and (7). The summation in Eq. (9) is taken over all the experimental motifs and |Q^
 MathType@MTEF@5@5@+=feaafiart1ev1aaatCvAUfKttLearuWrP9MDH5MBPbIqV92AaeXatLxBI9gBaebbnrfifHhDYfgasaacPC6xNi=xH8viVGI8Gi=hEeeu0xXdbba9frFj0xb9qqpG0dXdb9aspeI8k8fiI+fsY=rqGqVepae9pg0db9vqaiVgFr0xfr=xfr=xc9adbaqaaeGacaGaaiaabeqaaeqabiWaaaGcbaGafmyuaeLbaKaaaaa@2D0E@ - *Q*(*m*(e))| is the sum of squares of differences between individual elements of weight matrices Q^
 MathType@MTEF@5@5@+=feaafiart1ev1aaatCvAUfKttLearuWrP9MDH5MBPbIqV92AaeXatLxBI9gBaebbnrfifHhDYfgasaacPC6xNi=xH8viVGI8Gi=hEeeu0xXdbba9frFj0xb9qqpG0dXdb9aspeI8k8fiI+fsY=rqGqVepae9pg0db9vqaiVgFr0xfr=xfr=xc9adbaqaaeGacaGaaiaabeqaaeqabiWaaaGcbaGafmyuaeLbaKaaaaa@2D0E@ and *Q*(*m*(e)). The knowledge of the experimental motif is incorporated to the consensus motif adaptively with the distance function used in *f*_3_. Further, the fitness *f*_1 _optimizes the specificity and sensitivity of the prediction of binders.

The elements in the PSSM of experimental motifs are set to values within the same range [0, 2^*θ*^-1] as before. The following procedure is adopted to determine the elements of *Q*(*m*(e)): a well tolerated amino acid at an anchor position of the motif receives the highest possible score of 2^*θ*^-1; the lowest score of zero is assigned to a non-tolerated residue; weakly tolerated residues and residues at secondary anchor positions receive of (2^*θ*^-1)/2; and all the other unknown positions receives a score of (2^*θ*^-1)/3.

### Performance Comparison

The binding scores of I-A^g7 ^experimental motifs were computed using Eq. (8) by assigning the following values for binding strengths: primary = 4, secondary = 2, and others = 1, and for anchor positions: well = 4, weak = 2, non-tolerated = -4, and unknown = 0. The experimentally determined motifs were used with peptide data in the guided-discovery of motifs.

We used AUC to compare performance of the proposed methods with earlier approaches [28,34] and experimental motifs [38-44]. Whether a peptide is a binder or a non-binder is determined by a threshold of the binding score. By varying this threshold, the ROC curve was plotted, from which AUC value was obtained. A comparison of performances of the methods is given in Figure 1.

In order to compare to the MEME method, only binders in the I-A^g7 ^training set were submitted to MEME motif discovery tool at the prediction server [71]. The motif of 9-mer length was obtained with the following options: zero or one motif per sequence, minimum and maximum width = 9. The performance accuracy of RANKPEP approach on the testing dataset was carried out by uploading the dataset to the online prediction server at [72] with a 4% binding threshold [34].

### Benchmark Datasets

The proposed self-discovery approach was tested on BM-Set1, i.e., HLA-DRB1*0401, which consists of one training set and 10 testing datasets and had been earlier used to benchmark a number of motif finding algorithms [25,26,32,73]. The performance of MOEA was compared with earlier methods [8,10,32,35].

The training set consisting of binders and non-binders was assembled as follows: an ensemble of 532 unique binding peptides were extracted from SYFPEITHI [44] and MHCPEP [63] databases and a set of 177 unique non-binders were extracted from the MHCBN database [20]. The datasets were pre-processed by removing peptides that did not allow a hydrophobic residue at P1 position of all putative 9-mer binding cores and unnatural peptides containing more than 75% alanine [32]. The preprocessed binder set has 456 unique peptides with a length distribution ranging from 9 to 30 amino acid residues.

Of the 10 testing datasets, 8 datasets were taken from the MHC-bench as described in [74]. The other 2 datasets were extracted from experiments described by Southwood [75] and Geluk [76]. An affinity of (IC_50 _= 1000 nM) was taken as the threshold for peptide binding as described in [75]. Homology reduction had been carried out on all datasets in order to reduce the chances of over-fitting due to the redundancy of datasets. The peptides in the non-redundant (NR) datasets had sequence similarities less than 90%. The number of binders and non-binders in the original and NR datasets are given in Table 5.

We tested our method on BM-Set2 comprising of 3 mouse alleles and 13 HLA alleles made available at [77]. These quantitative peptide datasets had been extracted from the IEDB at [78]. The number of binders and non-binders in each dataset is given in Table 7. The DRB3-0101 allele dataset was excluded from the benchmark dataset because of the significant imbalance between binders and non-binders (3 binders and 99 non-binders). With this dataset, we compared our method with [8,10,11,32,35].

### Parameters of MOEA

The range of positional scores was set with *θ *= 7. For each run of MOEA, the population size *M *= 500, crossover probability *p*_*c *_= 0.9, and mutation probability *p*_*m *_= 0.005 were used. The process was terminated after 300 generations as no significant improvement in the convergence was observed during the experimental trial sessions. The parameters of the fitness functions were empirically determined for optimum performance within the following ranges: *κ*_1 _= 1~2.5, *α*_1 _= 5.0–6.0, and *α*_2 _= 1.0–2.0. The parameters *κ*_1 _= 2.5, *α*_1 _= 6.0, and *α*_2 _= 2.0 were found to work well empirically for both datasets.

## Authors' contributions

MR and VB conceived the study; MR designed experiments and performed computational analysis; MR, BS, VB and LF wrote the manuscript. All authors read and corrected the manuscript.

## Supplementary Material

Additional file 1 MOEA derived matrices on I-A^g7 ^dataset. The two PSSM derived by using MOEA self-discovery and guided-discovery approaches are given in the Additional file 1. Click here for file

Additional file 2 Motif logos obtained for I-A^g7 ^from MOEA derived matrices. Figure 1 and Figure 2 illustrate motif logos derived from the alignments obtained from the MOEA guided-discovery and self-discovery approaches. The web server [79] was used to generate the motif logos as described in [68]. Click here for file
