# Supplementary material for: Predicting peptides binding to MHC class II molecules using multi-objective evolutionary algorithms
Source: BMC Bioinformatics. 2007 Nov 22;8:459. doi: 10.1186/1471-2105-8-459 (PMC2212666; doi:10.1186/1471-2105-8-459)
Supplement: Additional file 1 — MOEA derived matrices on I-Ag7 dataset. The two PSSM derived by using MOEA self-discovery and guided-discovery approaches are given in the Additional file 1. [file 1471-2105-8-459-S1.pdf]

MOEA-derived matrices

Guided by the experimental motifs:

A	C	D	E	F	G	H	I	K	L	M	N	P	Q	R	S	T	V	W	Y
103.8	114.9	26.26	44.96	35.6	68.66	91.87	41.37	88.79	70.58	15.82	41.34	52.41	39	114.6	108.5	93.48	42.49	84.77	42.66
76.76	41.44	65.38	19.28	60.54	19.49	123.2	42.52	70.85	41.92	67.74	26.91	44.52	64.74	92.26	78.48	82.16	77.21	39.82	75.3
78.4	23.62	43.07	53.57	52.8	42.56	55.54	116.3	69.28	58.38	21.44	47.98	73.83	97.63	110	101.9	19.78	90.6	42.33	79.81
120.3	39.11	40.35	42.42	78.59	19.4	42.55	105.8	43.75	93.4	22.52	54.89	110.2	30.31	106.2	98.11	70.85	83.91	105.1	43.54
34.34	43.49	47.04	78.3	39.37	43.5	83.24	42.21	40.77	66.96	22.25	84.21	54.41	40.32	58.29	107	41.85	110	41.3	57.45
44.73	42.14	63.01	3.29	44.4	35.94	39.31	39.85	70.17	74.13	42.79	92.1	43.06	76.39	95.15	42.8	67.5	124.4	76.89	61.1
107.3	38.71	84.71	4.14	76.94	86.31	44.76	77.22	41.4	48.91	54.5	47.86	103.6	69.76	90.08	56.63	42.7	72.44	44.02	122.4
71.19	43.75	90.99	37.82	42.3	24.68	79.13	64.54	101.8	78.23	30.92	47.73	103.3	50.96	41.49	55.8	31.99	98.81	115.9	92.3
114.6	59.31	101	43.54	43.54	102.3	41.93	98.59	95.41	71.62	47.86	43.34	31.16	24.58	119.6	42.49	32.49	42.53	35.59	92.04

Self-discovered matrix:

A	C	D	E	F	G	H	I	K	L	M	N	P	Q	R	S	T	V	W	Y
59.2	87.7	5.95	11	17.7	44.4	15.9	27.5	69.8	69	29.9	45.6	119	126	126	111	77	15.3	97.2	73.5
1.11	62.1	102	1.27	2.24	0.43	127	11.2	47	116	48.8	5.56	80.7	60.7	104	125	41.6	68.8	45.5	127
29.7	8.38	22.4	7.46	19.4	94.5	52.8	116	3.42	124	45.7	36	75.2	56.6	116	117	95.4	104	27.9	85
75.8	55.2	25.6	57.3	53	0.93	18.8	91.6	41.7	104	32.3	79	111	4.65	123	115	34.4	88.7	104	24
39.6	107	46.2	8.08	87.6	94.4	45.6	116	2.18	92.3	98.6	111	69	68.4	103	96.9	76.6	74.2	2.22	24.6
97.9	9.76	118	31.6	3.75	109	26	6.92	10.7	78.1	29	91.2	110	22.4	85.9	24.4	92.5	126	85	0.07
114	104	32.9	46.8	20.3	106	50.6	125	15.1	54.2	16	17.1	86.7	80.7	30.1	127	20	102	6.28	117
77.4	126	23.9	9.21	58.5	24.9	126	93.1	59	98.1	78.6	37.2	97.9	5.06	120	78	64	126	121	127
116	19.5	57.4	0.75	38.4	69.7	95.8	118	89.3	17.7	1.44	112	21.2	2.06	91.9	51.9	13.8	85.3	21.3	104
