# Supplementary material for: Predicting peptides binding to MHC class II molecules using multi-objective evolutionary algorithms
Source: BMC Bioinformatics. 2007 Nov 22;8:459. doi: 10.1186/1471-2105-8-459 (PMC2212666; doi:10.1186/1471-2105-8-459)

Figure 1: illustrates the motif logo derived from the alignment obtained by the MOEA motif guided by the experimental motifs.

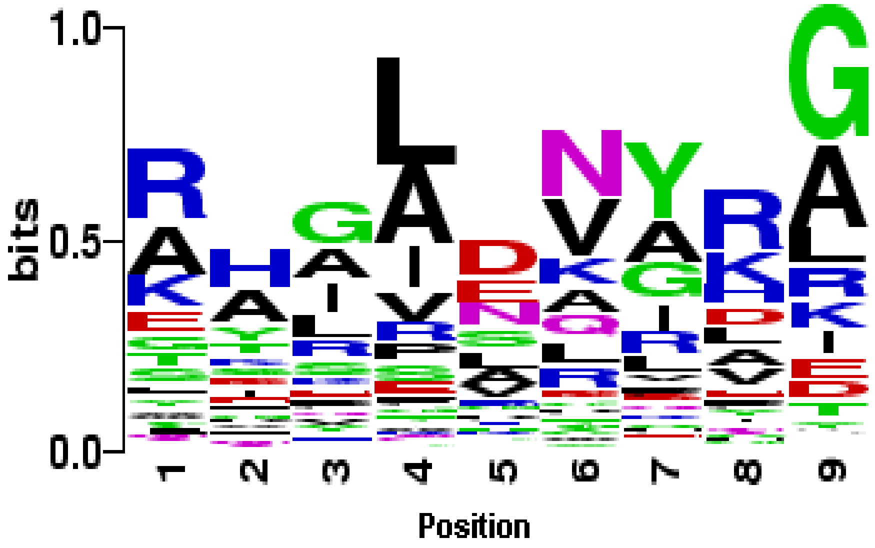

Figure 2: illustrates the motif logo derived from the alignment obtained by the self-discovered MOEA motif.

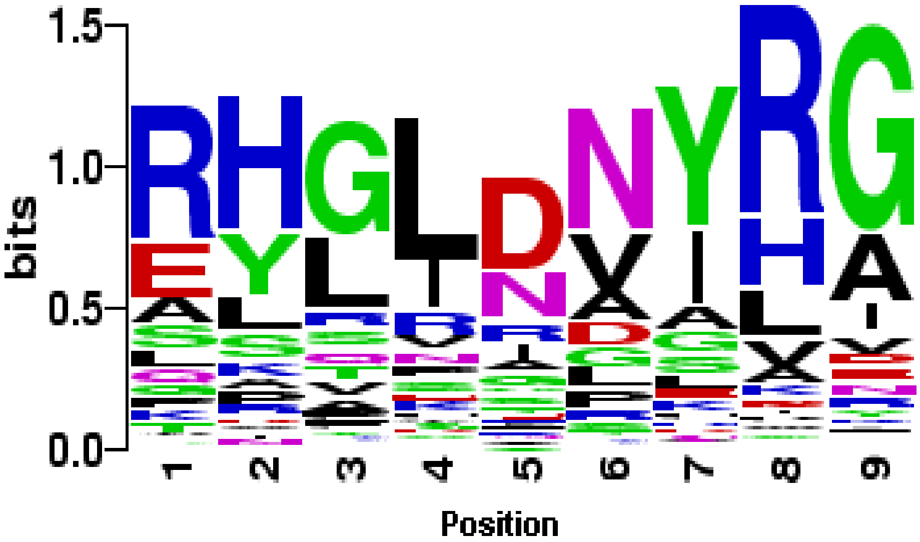

Supplement: Additional file 2 — Motif logos obtained for I-Ag7 from MOEA derived matrices. Figure 1 and Figure 2 illustrate motif logos derived from the alignments obtained from the MOEA guided-discovery and self-discovery approaches. The web server [79] was used to generate the motif logos as described in [68]. [file 1471-2105-8-459-S2.pdf]
